# Unanticipated Adverse Events With Tirzepatide: Three Cases Underscoring the Importance of Postmarketing Monitoring

**DOI:** 10.1210/jcemcr/luaf195

**Published:** 2025-08-29

**Authors:** Maria Colorado, Jose Gomez Miranda, Carlos E Arias-Morales

**Affiliations:** Department of Medicine, St. Barnabas Hospital, Bronx, NY 10457, USA; School of Medicine, City University of New York, Bronx, NY 10457, USA; School of Medicine, University of El Salvador, San Salvador 1101, El Salvador; Department of Medicine, St. Barnabas Hospital, Bronx, NY 10457, USA; School of Medicine, City University of New York, Bronx, NY 10457, USA

**Keywords:** tirzepatide, postmarketing surveillance, drug safety, adverse events

## Abstract

Tirzepatide (Zepbound), a novel dual glucose-dependent insulinotropic polypeptide and glucagon-like peptide-1 receptor agonist, demonstrates an additive effect, significantly enhancing insulin secretion compared to administering each hormone separately. The case reports presented here, detailing palpitations, musculoskeletal pain, and headaches following tirzepatide administration, raise important questions about the drug's postmarketing safety profile.

## Introduction

Tirzepatide, a novel dual agonist targeting glucose-dependent insulinotropic polypeptide (GIP) and glucagon-like peptide-1 (GLP-1) receptors marketed as Zepbound by its manufacturer Eli Lilly and Company, has shown significant efficacy in managing type 2 diabetes mellitus (T2DM) and obesity, as demonstrated in key clinical trials such as SURPASS [1] and SURMOUNT [2]. These studies, along with postmarketing surveillance [3], have largely established a safety profile characterized primarily by gastrointestinal adverse events. The case reports in this manuscript detail a series of patients who experienced distinct and potentially novel adverse reactions following the administration of tirzepatide, including palpitations, musculoskeletal pain, and headaches. These cases underscore the ongoing need for vigilance in postmarketing surveillance and raise important questions regarding the full spectrum of tirzepatide's tolerability. Here, we examine these case reports, place them within the broader context of tirzepatide's safety as established by clinical trials, and explore their implications for patient care and future research.

## Case Presentations

### Case 1

A 38-year-old female with a history of polycystic ovary syndrome and class I obesity presented to the clinic after reaching a plateau in her weight loss journey. She had been treated with the maximum weekly dose of semaglutide for 2 years, achieving approximately 10% weight loss from her baseline. The patient was switched to tirzepatide 5 mg subcutaneously weekly. The next day, she presented with palpitations, chest discomfort, and generalized myalgia following the first dose of tirzepatide.

### Case 2

A 37-year-old male with class I obesity, treated with tirzepatide 10 mg subcutaneously weekly, reported new-onset neck and back pain, and morning headaches, after his tirzepatide dose was increased from 7.5 to 10 mg.

### Case 3

A 43-year-old male with T2DM, class II obesity, hyperlipidemia, hypertension, and benign prostatic hyperplasia presented with acute complaint of bilateral neck and shoulder pain accompanied by morning headaches after initiating tirzepatide therapy. His current medications included tirzepatide 2.5 mg subcutaneously weekly, rosuvastatin 10 mg daily, amlodipine 10 mg daily, and tamsulosin 0.4 mg daily.

## Diagnostic Assessment

### Case 1

Less than 24 hours after the switch to tirzepatide, the patient reported paroxysmal palpitations that woke her from sleep, with a self-recorded heart rate of 130 beats per minute, accompanied by chest discomfort, generalized myalgia, and increased thirst, lightheadedness, and a home blood pressure reading of 90/58 mm Hg. Upon clinic presentation the next morning, her blood pressure was 92/65 mm Hg and heart rate was 110 beats per minute. An evaluation for other causes of tachycardia, including anemia, thyroid dysfunction, infection, and medication interactions, was unremarkable. No prior cardiovascular or autonomic symptoms were reported. The temporal relationship to tirzepatide initiation raised concern for a drug-related effect.

### Case 2

The patient experienced new-onset neck and back pain and morning headaches within 24 hours after tirzepatide administration. These symptoms were most pronounced on the 48 to 72 hours postinjection and resolved spontaneously. He also noted that injecting the medication in his abdominal area exacerbated his symptoms, while thigh injections were better tolerated. There was no evidence of trauma, infection, or structural spine pathology. The timing of symptom onset postinjection and resolution without intervention other than a change in injection site suggested a drug-related cause.

### Case 3

The patient reported morning headaches and bilateral neck and shoulder myalgia that began approximately 24 hours after initiating tirzepatide injections. Symptoms were most severe 72 hours after injection. Other etiologies such as tension headache, hypertension, or medication side effects were considered but not supported by clinical or laboratory findings. The symptom pattern recurring after injection supported a temporal association with tirzepatide.

## Treatment

### Case 1

The patient's medication was initially switched from semaglutide 2.4 mg to tirzepatide 5 mg subcutaneously weekly. The patient received only 1 dose of tirzepatide 5 mg and was switched back to semaglutide 2.4 mg subcutaneously weekly injections.

### Case 2

The patient continued tirzepatide at his current dose (10 mg subcutaneously weekly) and continued to experience the symptoms, but they were tolerable and did not prompt discontinuation of the medication.

### Case 3

The patient was advised to increase fluid intake and change the injection site. He reported partial relief with acetaminophen.

## Outcome and Follow-up

### Case 1

Her palpitations and myalgia improved after increased fluid intake and cessation of tirzepatide. All reported symptoms resolved within 1 week. No recurrence was observed after resuming semaglutide.

### Case 2

The patient continued to experience mild myalgia and morning headaches following weekly injections but these were less pronounced with thigh injection site. He also continued tirzepatide at the current dose of 10 mg because he found the symptoms tolerable.

### Case 3

At a follow-up visit, his symptoms had resolved after switching the injection site to the arm. The tirzepatide dose was subsequently increased to 5 mg weekly without recurrence of symptoms.

## Discussion

Peptide hormones are vital for metabolic regulation, maintaining balance in sugars, lipids, and energy. Beyond insulin, incretin hormones such as GLP-1 and GIP are key players. Tirzepatide, the first dual GIP/GLP-1 receptor agonist (RA), received US Food and Drug Administration (FDA) approval for the treatment of T2DM in May 2022 and for chronic weight management in November 2023 [4].

**Figure 1.**
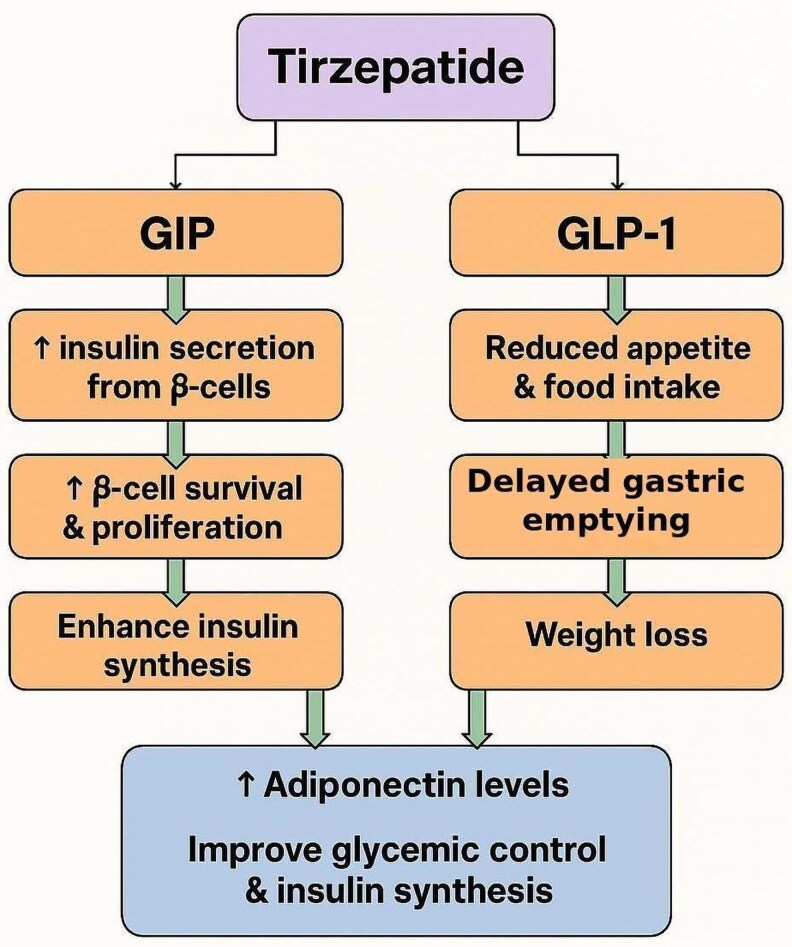
Diagram of the mechanism of action of tirzepatide.

Coadministration of GIP and a GLP-1 RA in healthy individuals has an additive effect, significantly enhancing insulin secretion compared to administering each hormone separately (Fig. 1). This combined infusion also significantly suppresses glucagon secretion, an effect not observed with individual GIP or GLP-1 RA administration beyond glucose-induced suppression. However, in individuals with T2DM, short-term coadministration (4-6 hours) of GIP and GLP-1 RA does not elicit a more significant insulin response than GLP-1 RA administration alone [1].

The SURPASS-5 clinical trial, which investigated the effect of tirzepatide added to titrated insulin glargine in patients with T2DM, provided valuable insights into its tolerability. Gastrointestinal adverse events were the most frequently reported treatment-emergent side effects in the tirzepatide groups. These included diarrhea, nausea, vomiting, and decreased appetite. Most of these gastrointestinal events were mild to moderate in severity, and the incidence of new events decreased over time across all tirzepatide dosage groups.

The incidence of hypoglycemia (blood glucose <70 mg/dL [SI: < 3.9 mmol/L]) and severe hypoglycemia (blood glucose <40 mg/dL [SI: < 2.2 mmol/L]) ranged from 14.2% to 19.3% in tirzepatide groups vs 12.5% in placebo. Three adjudication-confirmed major adverse cardiovascular events, a composite endpoint of death from cardiovascular or undetermined causes, myocardial infarction, stroke, and hospitalization for unstable angina, were reported within the patient population of this trial, where tirzepatide was added to titrated insulin glargine [5].

In the SURPASS-2 trial, which compared tirzepatide with semaglutide in patients with T2DM, a total of 13 deaths (0.7%) occurred. These deaths were distributed as follows: 4 in each tirzepatide group (5, 10, and 15 mg) and 1 in the semaglutide group. An independent adjudication committee determined that none of these deaths were related to either tirzepatide or semaglutide. Notably, 5 deaths were attributed to COVID-19, and 1 cardiovascular death was associated with suspected COVID-19. Other causes of death included cardiovascular events (4 cases, distributed across the tirzepatide groups) and indeterminate causes (2 cases, 1 each in the 5 mg and 10 mg tirzepatide groups). Two patients with cardiovascular deaths had no prior cardiovascular risk factors other than T2DM.

Adjudicated pancreatitis cases were reported in both tirzepatide (2 cases each in the 10 mg and 15 mg groups) and semaglutide (3 cases) recipients, with no cases considered serious. Changes in serum alanine aminotransferase ranged from -22% to -30% with tirzepatide and -22% with semaglutide, and changes in aspartate aminotransferase ranged from -9% to -14% with tirzepatide and -9% with semaglutide. Cholelithiasis was reported in 4 patients in each tirzepatide group and 2 in the semaglutide group. No clinically significant changes in calcitonin levels or cases of medullary thyroid carcinoma were observed. Two cases of diabetic retinopathy were reported, both in the tirzepatide 10 mg group.

Hypersensitivity reactions occurred in 1.7% to 2.8% of tirzepatide recipients and 2.3% of semaglutide recipients. Injection site reactions, generally mild to moderate, were reported in 1.9% to 4.5% of tirzepatide recipients and 0.2% of semaglutide recipients. Similar results were observed for glycated hemoglobin levels, body weight, and achievement of glycated hemoglobin and weight loss targets [6].

In the SURMOUNT-1 trial, which evaluated tirzepatide for obesity treatment, similar adverse event profiles were observed, though specific rates varied. The overall safety findings in both trials highlight the tolerability of tirzepatide, with gastrointestinal side effects being the most common and generally mild to moderate [7].

The case reports presented, detailing palpitations, musculoskeletal pain, and headaches following tirzepatide administration, raise important questions about the drug's postmarketing safety profile. While the pivotal clinical trials, namely the SURPASS and SURMOUNT series, established the efficacy of tirzepatide in managing T2DM and obesity, respectively, they also provided a foundation for understanding its tolerability. Predominantly, these trials highlighted gastrointestinal adverse events as the most common, including nausea, vomiting, diarrhea, and decreased appetite. However, the emergence of distinct symptoms, such as those described in these cases, necessitates deeper exploration.

The cardiovascular observation of palpitations in the first case is particularly noteworthy. Significant palpitations are not commonly reported as a side effect. The documented tachycardia and accompanying hypotension in this patient imply a potential idiosyncratic reaction that may be influenced by individual patient factors, such as preexisting conditions or concurrent medications, although none was noted in this instance. The transition from semaglutide, another GLP-1 RA, adds a layer of complexity to the situation. Although a direct interaction is unlikely, the possibility of a withdrawal effect or unusual cross-reactivity cannot be entirely dismissed.

The musculoskeletal complaints of myalgia and headaches, as seen in the second and third cases, also warrant attention. Although musculoskeletal discomfort was reported in some trial participants, the intensity, persistence, and specific patterns observed in these cases are notable. The correlation between increased tirzepatide dosage and symptom exacerbation in the second case suggests a dose-dependent effect, potentially related to the drug's pharmacodynamics. The resolution of symptoms following a change in injection site in the third case points to a potential localized reaction, possibly related to tissue irritation or inflammation at the injection site. This highlights the importance of patient education regarding proper injection techniques and site rotation.

In all 3 cases, potential alternative causes were evaluated and deemed unlikely based on history, physical examination, and basic workup. This strengthens the plausibility of tirzepatide as the likely contributor to the reported symptoms.

The lack of comparable reports in the published literature, along with the infrequent occurrence of these specific symptoms during clinical trials, highlights the importance of postmarketing surveillance. The FDA's Adverse Event Reporting System is essential for capturing and analyzing these reports, offering valuable insights into drug safety in real-world settings. The manufacturer's collaboration with medical providers, including conversations with medical liaisons, reflects a strong commitment to monitoring and addressing potential safety issues.

## Learning Points

- Although clinical trials for tirzepatide primarily identified gastrointestinal issues as the main adverse events, postmarketing surveillance is revealing potentially novel side effects like palpitations and myalgia.
- Individual patient responses to tirzepatide can vary, and factors such as dosage changes and injection site may influence the occurrence and severity of certain adverse events.
- The observation of these distinct adverse events, not prominent in initial trials, underscores the critical role of ongoing postmarketing surveillance and adverse event reporting in fully characterizing a drug's safety profile in a broader patient population.
- These cases suggest potential adverse events associated with tirzepatide, including palpitations, myalgia, headache, and injection site-related pain. An adverse event report was submitted to the manufacturer, and the medical provider discussed these cases with the manufacturer's medical liaison. Notably, these symptoms were not reported during tirzepatide's clinical trials. This suggests that these may be postmarketing adverse events. A review of current literature revealed no similar case reports.

## Contributors

All authors made individual contributions to authorship. The contributions to the manuscript were equal among C.A.M., M.C., and J.G.M. All authors reviewed and approved the final draft.

## Data Availability

Data sharing is not applicable to this article as no datasets were generated or analyzed during the current study.
